# Accuracy of 3D real-time MRI temperature mapping in gel phantoms during microwave heating

**DOI:** 10.1186/s41747-024-00479-5

**Published:** 2024-08-14

**Authors:** Olaf Dietrich, Sergio Lentini, Osman Öcal, Pierre Bour, Thibaut L. Faller, Valéry Ozenne, Jens Ricke, Max Seidensticker

**Affiliations:** 1 Department of Radiology, LMU University Hospital, LMU Munich, Munich, Germany; 2 Department of Diagnostic and Interventional Radiology, Heidelberg University Hospital, Heidelberg, Germany; 3 Certis Therapeutics, Pessac, France; 4 Université de Bordeaux, CNRS, CRMSB, UMR 5536, IHU Liryc, Bordeaux, France

**Keywords:** Artifacts, Magnetic resonance imaging (interventional), Microwaves, Temperature, Phantoms (imaging)

## Abstract

**Background:**

Interventional magnetic resonance imaging (MRI) can provide a comprehensive setting for microwave ablation of tumors with real-time monitoring of the energy delivery using MRI-based temperature mapping. The purpose of this study was to quantify the accuracy of three-dimensional (3D) real-time MRI temperature mapping during microwave heating *in vitro* by comparing MRI thermometry data to reference data measured by fiber-optical thermometry.

**Methods:**

Nine phantom experiments were evaluated in agar-based gel phantoms using an in-room MR-conditional microwave system and MRI thermometry. MRI measurements were performed for 700 s (25 slices; temporal resolution 2 s). The temperature was monitored with two fiber-optical temperature sensors approximately 5 mm and 10 mm distant from the microwave antenna. Temperature curves of the sensors were compared to MRI temperature data of single-voxel regions of interest (ROIs) at the sensor tips; the accuracy of MRI thermometry was assessed as the root-mean-squared (RMS)-averaged temperature difference. Eighteen neighboring voxels around the original ROI were also evaluated and the voxel with the smallest temperature difference was additionally selected for further evaluation.

**Results:**

The maximum temperature changes measured by the fiber-optical sensors ranged from 7.3 K to 50.7 K. The median RMS-averaged temperature differences in the originally selected voxels ranged from 1.4 K to 3.4 K. When evaluating the minimum-difference voxel from the neighborhood, the temperature differences ranged from 0.5 K to 0.9 K. The microwave antenna and the MRI-conditional in-room microwave generator did not induce relevant radiofrequency artifacts.

**Conclusion:**

Accurate 3D real-time MRI temperature mapping during microwave heating with very low RMS-averaged temperature errors below 1 K is feasible in gel phantoms.

**Relevance statement:**

Accurate MRI-based volumetric real-time monitoring of temperature distribution and thermal dose is highly relevant in clinical MRI-based interventions and can be expected to improve local tumor control, as well as procedural safety by extending the limits of thermal (*e.g*., microwave) ablation of tumors in the liver and in other organs.

**Key Points:**

- Interventional MRI can provide a comprehensive setting for the microwave ablation of tumors.
- MRI can monitor the microwave ablation using real-time MRI-based temperature mapping.
- 3D real-time MRI temperature mapping during microwave heating is feasible.
- Measured temperature errors were below 1 °C in gel phantoms.
- The active in-room microwave generator did not induce any relevant radiofrequency artifacts.

**Graphical Abstract:**

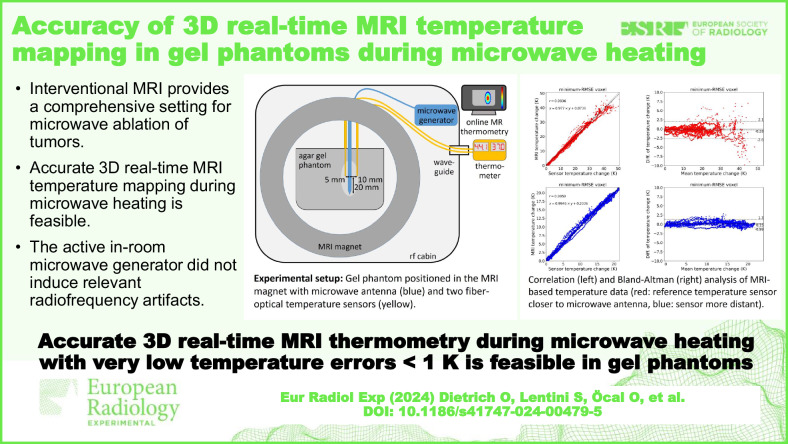

## Background

Interventions performed under magnetic resonance imaging (MRI) guidance have several advantages when compared to those performed under computed tomography or ultrasound guidance, including multiplanar acquisitions, superior image contrast for lesion visualization, and absence of ionizing radiation exposure [1–4]. In particular, percutaneous biopsies and treatment of liver tumors can substantially benefit from MRI guidance [5–10].

Local ablation of liver tumors has become established in selected patients (*e.g*., those with oligofocal metastases or oligometastatic disease) in various entities [11–16] and is playing an increasing role alongside resection. Compared to resection, local ablation has the advantage of lower morbidity with comparable effectiveness [17, 18]. The most widespread is local ablation using thermal methods, in particular radiofrequency (RF) ablation or microwave ablation [19–22]. Local heating of the tumor tissue (including a safety margin) to around 100 °C devitalizes the tumor. However, local recurrence rates range from around 11.9% for microwave ablation to 16.8% for RF ablation [23]. Further on, the applicability of thermal ablation is limited by the size of the target lesion (maximum of about 3 cm) and the presence of adjacent large cooling vessels, as well as by proximity to heat-vulnerable structures such as main bile ducts, stomach, duodenum, colon, or heart, with a minimal required distance of 1 cm [24, 25]. In order to optimize the efficacy, as well as safety of local thermal ablation procedures, real-time *in vivo* thermometry is warranted to verify complete ablation, as well as to indicate critical heating of heat-vulnerable structures.

Interventional MRI can provide a comprehensive setting for microwave ablation treatment of liver tumors that comprises high-contrast lesion identification, real-time fluoroscopic image guidance of the microwave applicator to the target, and—unique to this modality—the ability to non-invasively monitor and visualize the energy delivery using quantitative real-time MRI-based temperature mapping techniques [26–28]. Obviously, for clinical applications, the accuracy and reliability of such MR thermometry approaches is highly relevant with regard to the efficacy and safety of the procedure.

A typical MRI-based microwave ablation treatment setup consists of the MRI scanner, the MRI protocols for real-time applicator guidance and MRI thermometry, the (in-room) microwave generator and applicator, as well as the MRI thermometry real-time visualization software. In general, these components are provided by different manufacturers, and in particular, the MRI protocol depends on specific local requirements. Several implementation details, system specifications, and interactions between these components will influence the resulting accuracy of MRI thermometry data. Examples are the signal-to-noise ratio of the acquired phase data, the influence of residual RF noise from the (in-room) microwave generator (including the microwave cable and applicator), as well as susceptibility artifacts around the microwave antenna that are worsened by gas bubbles due to tissue (over)heating. No detailed a priori knowledge of these factors is available, and the resulting temperature accuracy cannot be provided by the manufacturers of the individual components.

Hence, the purpose of this study was to quantify the accuracy of three-dimensional (3D) real-time MRI temperature mapping during microwave heating *in vitro* by comparing MRI thermometry data to reference data measured by fiber-optical thermometry.

## Methods

In this study, 3D real-time MRI thermometry of gel phantoms was performed during local microwave heating, and the temperatures determined by MRI were compared to conventional (fiber-optical) thermometry as described in detail below.

### Phantoms

All measurements for this study were performed in two agar-based gel phantoms with dimensions of about 17 × 24 × 14 cm³ corresponding to approximately 6 L. The phantoms were made from (consumer-grade) agar with a concentration of 30 g/L. One phantom was based on pure water as solvent, and the second one was based on physiological (0.9%) NaCl solution. Before the measurements, the phantoms were stored for at least 3 h in the MRI RF cabin to make sure that they were at room temperature (21 °C). In total, ten phantom experiments were performed (four experiments in the saline phantom and six experiments in the water phantom).

### Microwave device and heating protocol

**Fig. 1:**
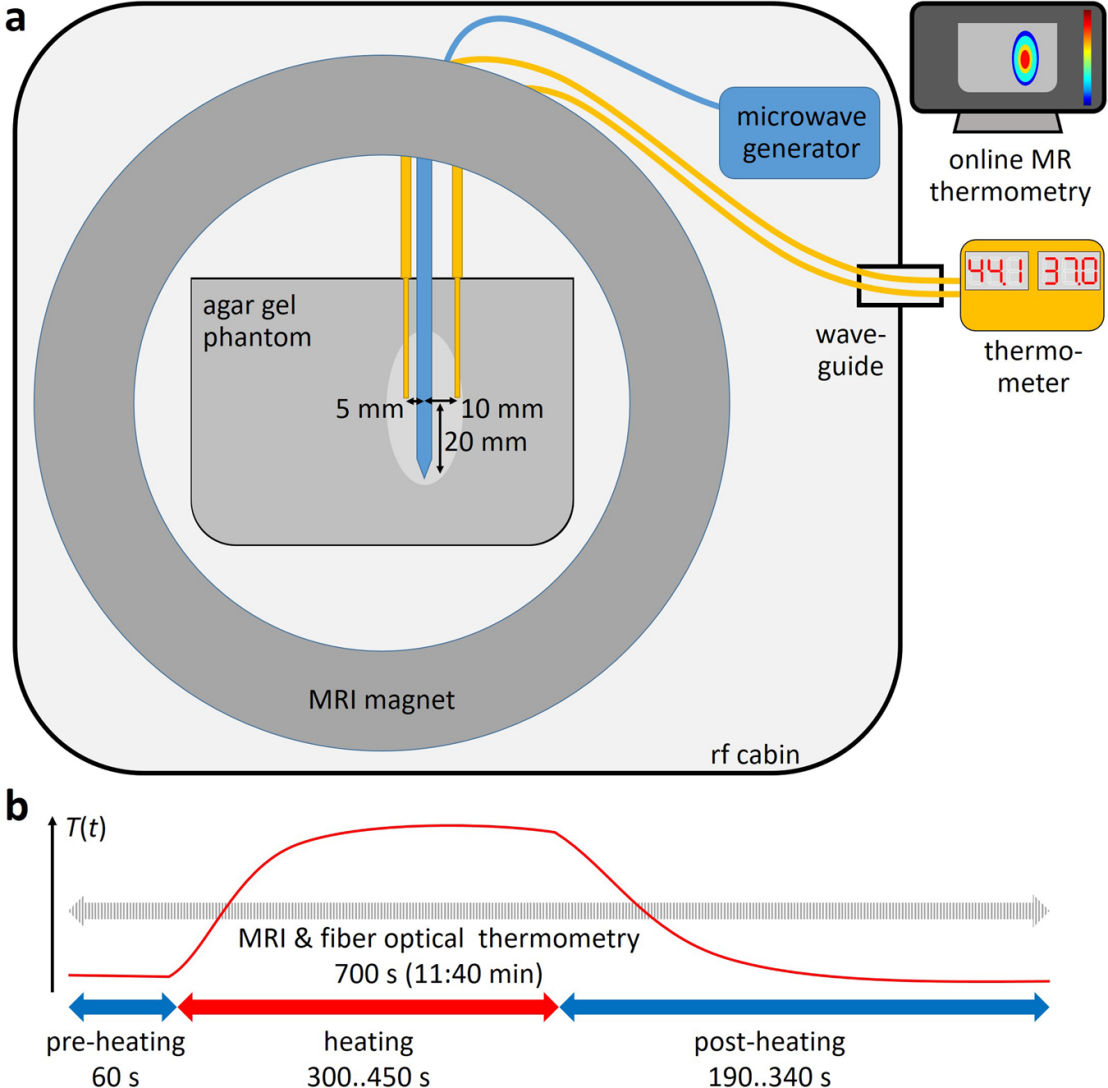
Experimental setup and heating protocol. **a** The gel phantom was positioned in the MRI magnet with a microwave antenna (blue) and two fiber-optical temperature sensors (yellow); the MRI-conditional microwave generator remained in the RF cabin; the thermometer unit was outside. **b** Time course of heating experiments showing the temperature change *T* over the time *t*

The experimental setup and the heating procedure are illustrated in Fig. 1. Local heating of the phantom was achieved with a (Conformité Européene (CE)-marked and USA Food and Drug Administration (FDA)-approved) MRI-conditional microwave system (AveCure, MedWaves, San Diego, CA, USA) with a microwave power of 40 W at frequencies of 902–928 MHz. A 14-gauge microwave antenna (AveCure 14 Gauge Probe Medium Antenna, MedWaves, San Diego, CA, USA; CE-marked and FDA-approved) with a needle with a usable length of 14 cm and diameter of 2 mm (and without needle cooling) was inserted vertically into the gel phantom to a depth of 8–10 cm with sufficient distance (> 2 cm) from the phantom boundaries. The antenna was designed to produce an approximately elliptical heating field with a length (along the antenna) of about 4 cm. It was reinserted into previously non-heated parts of the phantoms for each of the ten experiments.

The heating protocol consisted of a 60-s baseline preheating period, followed by 300 s of heating in the saline-based phantom or 450 s of heating in the water-based phantom, and, finally, a 190–340 s post-heating period for cool-down. The total experiment duration was 700 s. The microwave generator was set to an intended (but never actually reached) target temperature measured at the antenna of 120 °C in the water phantom and 60 °C (80 °C in one case) in the saline phantom.

### Fiber-optical thermometry device

For (conventional) reference temperature monitoring, a thermometer with two fiber-optical temperature sensors (Neoptix, Québec, Canada) was used; the sensors (outer diameter 1.15 mm, sensitive area diameter 300 µm) were also inserted vertically into the phantom approximately 5 mm and 10 mm distant from the microwave antenna. The temperature-sensitive tips of the sensors were aligned with the position of maximum heating about 2 cm above the tip of the antenna. Fiber-optical temperature data were acquired with a frequency of 1 Hz and saved to a text file. The absolute accuracy of the thermometer was specified as ± 1.0 °C, with manufacturer-provided calibration readings of 49.7/99.8 °C (sensor 1) and 49.6/99.8 °C (sensor 2) at control temperatures of 50.0/100.0 °C.

### MRI setup and acquisition

The experimental setup consisting of the phantom, the microwave antenna, and the fiber-optical temperature sensors was positioned at the isocenter of a 1.5-T whole-body MRI system (Magnetom Aera, Siemens Healthineers, Erlangen, Germany); the microwave generator was also in the RF cabin, but approximately 3 m distant from the magnet isocenter. The thermometer unit was positioned outside of the RF cabin. An 11-cm loop coil and 12 coil elements of the built-in spine array were used for signal reception. Proton-resonance-frequency-based MRI thermometry [29–31] was performed with a prototype gradient-echo single-shot echo-planar imaging (EPI) sequence, which was modified to reconstruct phase images in addition to the standard magnitude images from the multichannel raw data. The EPI thermometry pulse sequence acquired 25 coronal slices (3-mm slice thickness) with an in-plane resolution of 2.2 × 2.2 mm² and a field of view of 280 × 280 mm². The temporal resolution for each block of 25 slices, *i.e*., the repetition time, was 2 s, the echo time was 18 ms, and the flip angle was 90°. Partial-Fourier (factor 6/8) and parallel imaging (factor 2) were used to accelerate the acquisition; the receiver bandwidth was 1,628 Hz/pixel.

### Temperature map estimation

The MRI measurements were performed for 700 s resulting in 350 individual 3D temperature measurements over time. The MRI phase and magnitude data were transmitted in real-time to a workstation where temperature maps were calculated and displayed online using the CE-marked software “Certis Solution”, version 1.2.0 (Certis Therapeutics, Pessac, France). The thermometry pipeline included an image-based temperature drift correction and noise suppression in addition to thermal dose calculation (thermal dose data were not used in this work). After each experiment, the generated 4-dimensional (3D volume plus time) temperature maps were exported for further analysis with Python 3.11.

### Data analysis

The two fiber-optical temperature sensors provided reference data sets consisting of a total of 7,000 = 10 (experiments) × 2 (sensors) × 350 (time points, temporally down-sampled to 0.5 Hz) data points. These reference thermometer data were normalized by subtracting the arithmetic mean of the first five data points to represent the temperature change, *T* (as measured by proton resonance frequency-MRI).

To characterize the heating processes, the reference data sets of the temperature sensors were evaluated with respect to the maximum temperature change over each experiment given in kelvin (K).

**Fig. 2:**
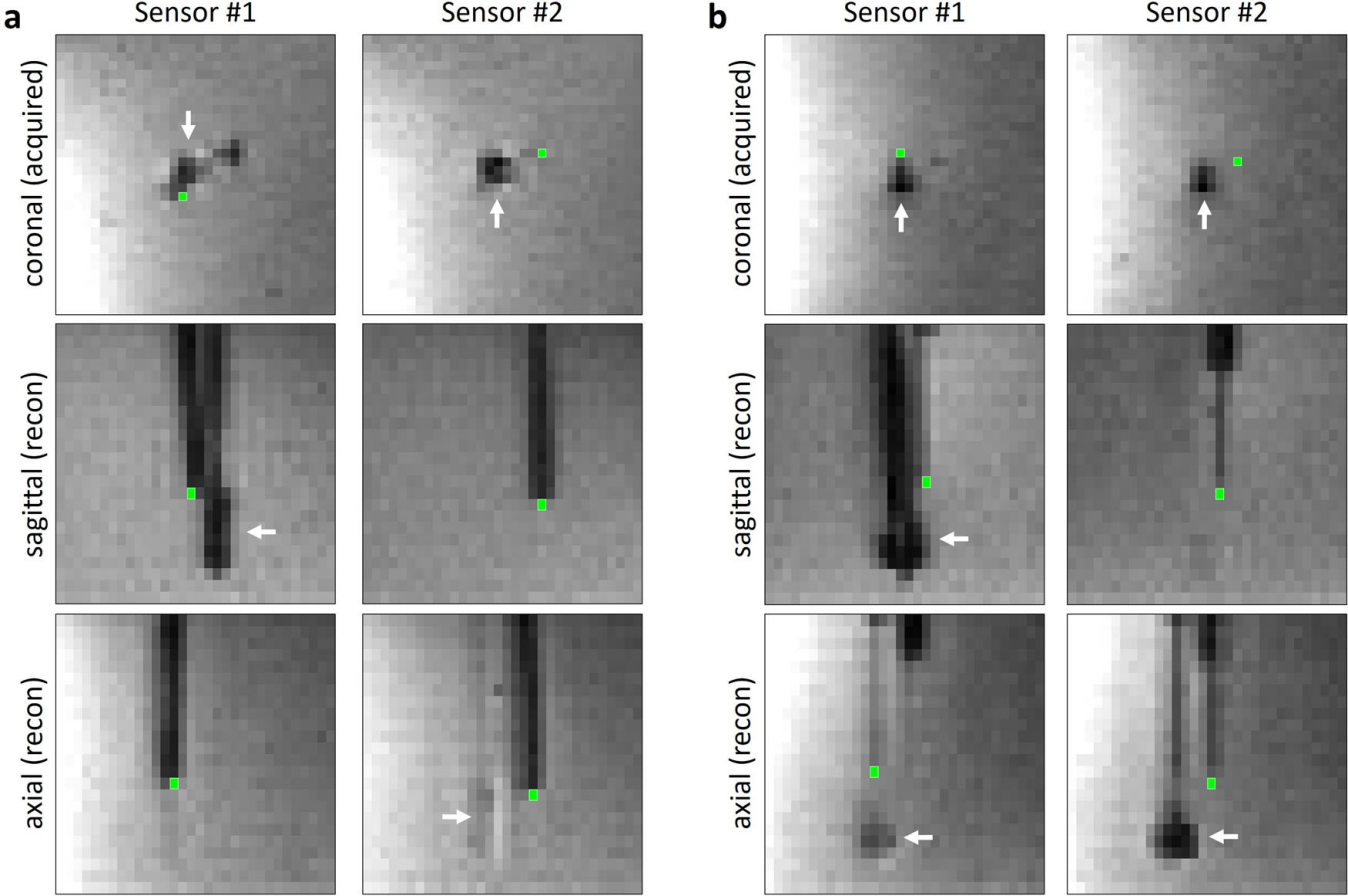
Selection of single-voxel ROIs (green rectangles) in magnitude echo-planar images at the tip of the two temperature sensors in two different experiments (**a**, **b**). The (artifact of the) microwave antenna is indicated by the white arrow

To assess the MRI-based thermometry maps, single-voxel regions of interest (ROIs) at the position visually corresponding to the tip of the fiber-optical temperature sensors were independently defined by three readers (two experienced radiologists and a physicist) based on the EPI magnitude images (Fig. 2). The temperature curves of the two sensors, *T*_sensor_(*t*), were then compared to the MRI temperature data at these ROIs, *T*_MRI_(*t*), by calculating the root-mean-squared (RMS) difference over the complete 700-s (*N* = 350 measurements) time course:

$$\Delta {T}_{{{{{\rm{RMS}}}}}}\,=\,\sqrt{\frac{1}{N}{\sum }_{i}^{N}{\left({T}_{{{{{\rm{MRI}}}}}}\left({t}_{i}\right)\,-\,{T}_{{{{{\rm{sensor}}}}}}\left({t}_{i}\right)\right)}^{2}\,}.$$

To account for the remaining uncertainty about the exact sensor tip location (and the volume over which the sensor integrates the gel temperature), 18 neighboring voxels around the original (reader-selected) ROIs (*i.e.*, a 3 × 3 × 3 cube without the eight corner voxels approximating an ellipsoid around the center voxel) were also evaluated, and the voxel with the minimum RMS difference was additionally selected for further evaluation. Due to the slightly anisotropic resolution, the maximum in-plane distance of the evaluated voxels was ± 2.2 mm (in each axis) and the maximum through-plane distance was ± 3.0 mm. The extent and frequency of the required shifts of this ROI-fitting process were collected and analyzed. For this “minimum root-mean-squared error” (minRMSE) voxel, we then also determined Δ*T*_RMS_, as well as the maximum (positive and negative) temperature differences over the time course:

$$\Delta {T}_{\max ,+}\,=\,{\max }_{{t}_{i}}\left({T}_{{{{{\rm{MRI}}}}}}\left({t}_{i}\right)\,-\,{T}_{{{{{\rm{sensor}}}}}}\left({t}_{i}\right)\right),$$

(describing the maximum deviation of the MRI thermometry measurement above the reference values), and:

$$\Delta {T}_{\max ,-}\,=\,{\max }_{{t}_{i}}\left[-\left({T}_{{{{{\rm{MRI}}}}}}\left({t}_{i}\right)\,-\,{T}_{{{{{\rm{sensor}}}}}}\left({t}_{i}\right)\right)\right]$$

(describing the maximum deviation of the MRI thermometry measurement below the reference values).

Results are presented as median values (interquartile range (IQRs)) of the temperature differences over all experiments.

Finally, the agreement between the sensor-derived reference data and the MRI-based temperature maps was assessed using linear regression analysis, Pearson correlation coefficients, correlation plots, and Bland–Altman plots [32].

## Results

**Fig. 3:**
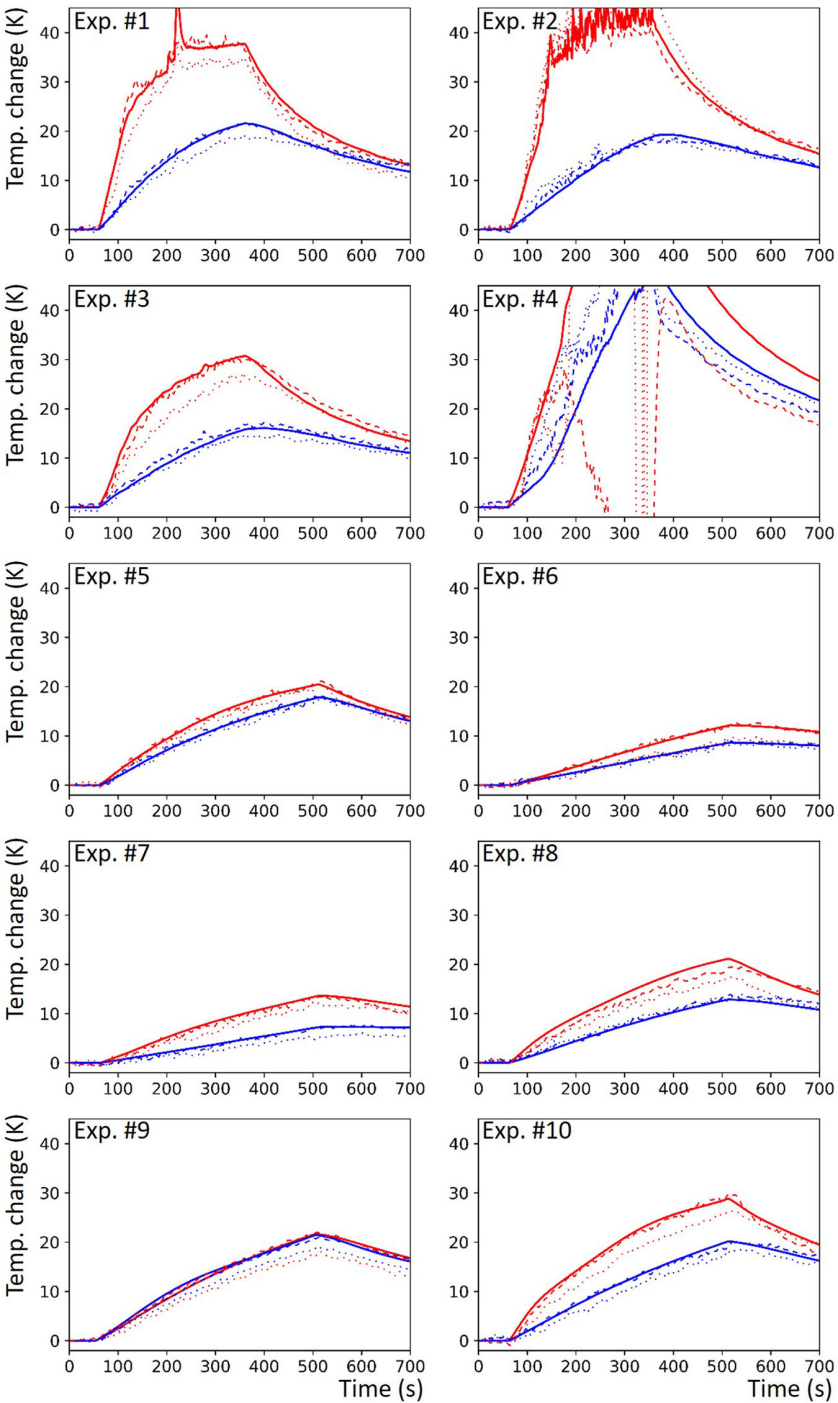
Measured time courses of temperature changes (relative to room temperature) from all ten experiments. Solid lines: reference thermometry with fiber-optical sensor; dotted lines: MRI temperature data from reader-selected ROIs (only data from reader #1 is shown here); dashed lines: MRI temperature data from minRMSE ROIs. Experiments #1 to #4 were performed in the saline-based gel phantom, and experiments #5–#10 in the water-based phantom. minRMSE, Minimum root-mean-squared error

The reference fiber-optical thermometry data of all experiments are shown in Fig. 3 (solid lines). The maximum temperature changes over each time course measured by the fiber-optical sensors ranged from 12.1 K to 73.9 K for the sensor closer to the microwave antenna (red line) and from 7.3 K to 48.3 K for the sensor more distant from the antenna (blue line). The median values (IQRs) over the ten experiments were 25.2 K (29.8 K) and 18.6 K (9.7 K), respectively.

**Fig. 4:**
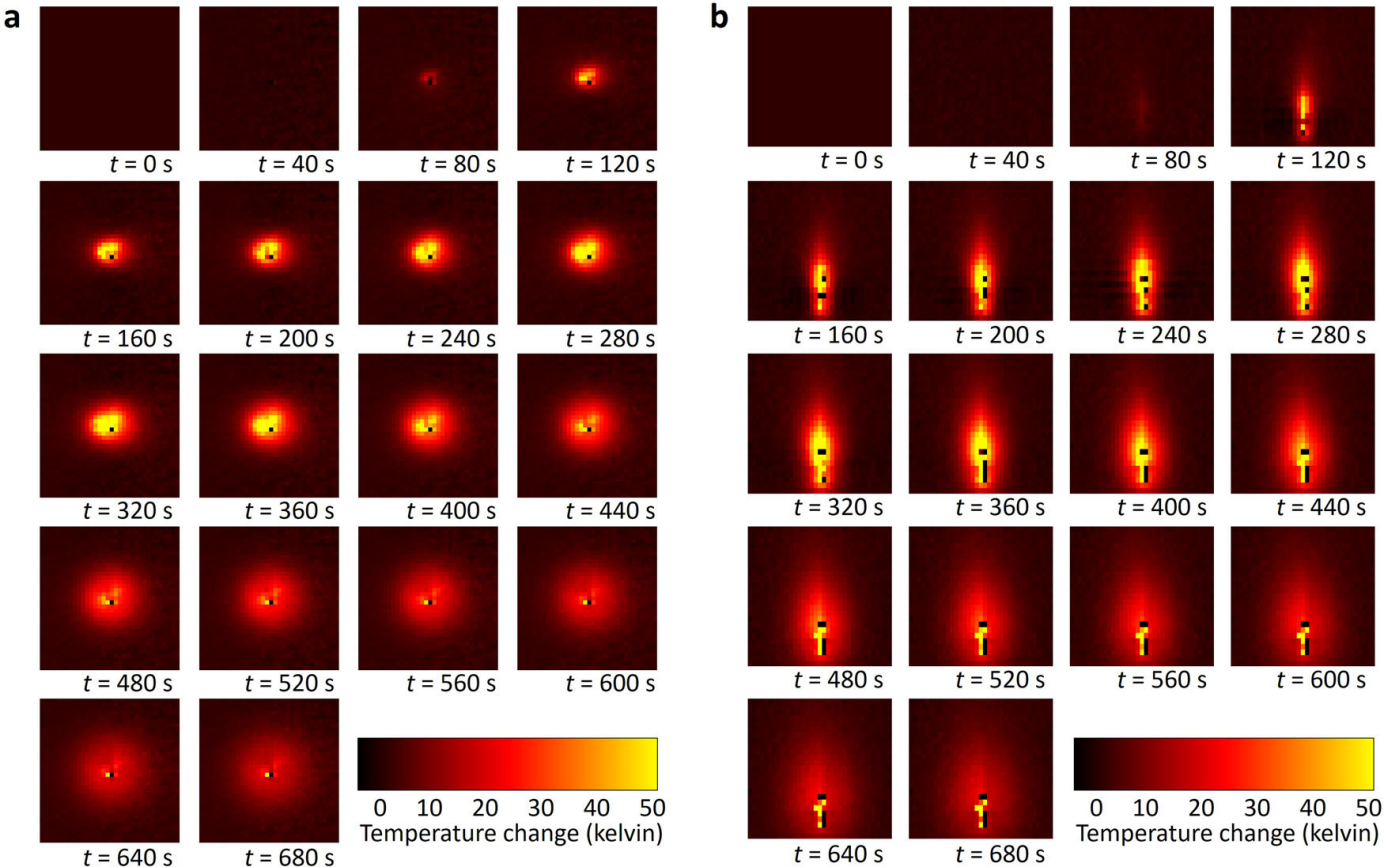
Four-dimensional maps of temperature changes (three spatial dimensions plus time, *t*, only every 20th-time point is shown) of experiment #2 (see Fig. 3): (**a**) coronal sections as acquired, (**b**) reconstructed axial sections. Microwave heating was performed from *t* = 60 s to 360 s. At black voxels, no temperature calculation could be performed due to insufficient signal-to-noise ratio

A typical example of the acquired four-dimensional MRI temperature maps is shown in Fig. 4. Heating starts at *t* = 60 s and is initially visible in the next shown map at *t* = 80 s. Heating stops at *t* = 360 s; after that, the thermal energy stored in the gel is conducted away from the antenna. We did not observe any visual change of background noise (*e.g*., due to RF artifacts) during the activation of the microwave generator. The corresponding MRI thermometry time courses for the voxel ROIs of Reader 1 are shown in Fig. 3 as dotted lines, and display qualitatively the same behavior as the reference sensor measurements.

One of the ten measurements (experiment #4 in Fig. 3) showed very atypical MRI thermometry data; in this case, the gel phantom had been overheated and a larger gas bubble of vaporized gel had appeared, in which MRI thermometry could not be performed. This experiment was therefore excluded from further analysis. In the remaining nine experiments, the maximum temperature changes measured by the fiber-optical sensors ranged from 12.1 K to 50.7 K for the sensor closer to the microwave antenna and from 7.3 K to 21.6 K for the sensor more distant from the antenna. The median values (IQRs) of the maximum temperature change over the remaining nine experiments were 21.7 K (22.2 K) and 17.8 K (10.1 K), respectively.

**Fig. 5:**
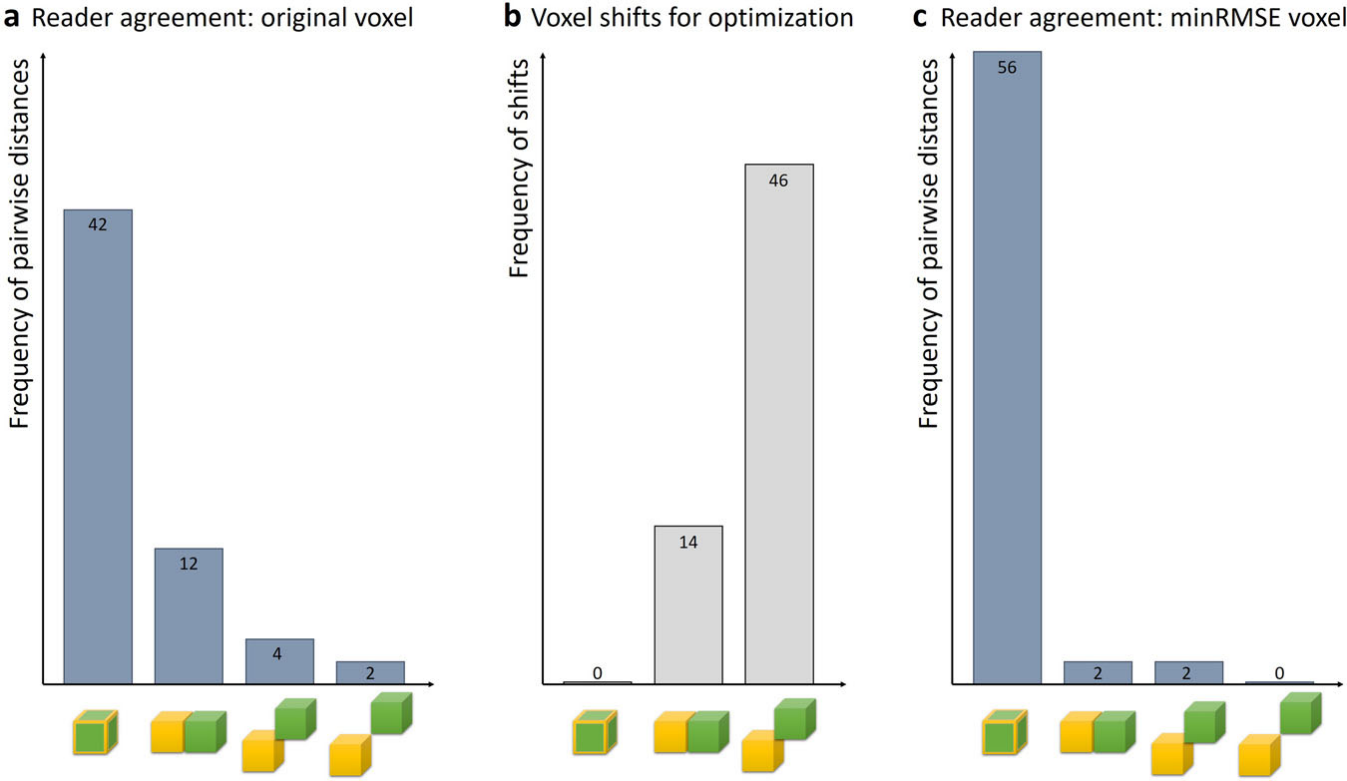
**a** Reader agreement when positioning the single-voxel ROIs (evaluated pairwise, resulting in 60 pairs = three pairs of readers × two ROIs × ten experiments). **b** Required shifts to minimize the temperature difference between sensor measurements (reference) and MRI thermometry (60 shifts for three readers × two ROIs × ten experiments). **c** Pairwise (reader) agreement of ROIs after determination of the (neighboring) minRMSE voxel. The distance is indicated below the horizontal axis (from left to right: identical, *i.e*., perfect agreement, next neighbor, planar diagonal neighbor, and spatially diagonal neighbor). minRMSE, Minimum root-mean-squared error

The agreement of the single-voxel ROI positioning of the three readers is illustrated in Fig. 5a. Of 60 pairwise comparisons (three pairs of readers × two ROIs × ten experiments), 42 ROIs were defined identically, 12 were defined in neighboring voxels, four in planar-diagonally positioned voxels, and two in spatially-diagonally voxels; no ROIs were further apart than that.

**Fig. 6:**
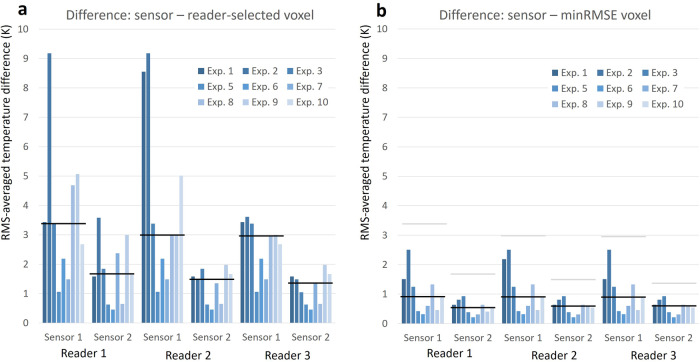
RMS-averaged temperature differences between reference sensor data and MRI thermometry. RMS, Root-mean-squared

The RMS-averaged temperature differences between reference (sensor) data and MRI-thermometry data are listed in Table 1 (left half) and shown in Fig. 6a. In the original reader-selected voxels, the median values (IQRs) of RMS-averaged temperature differences over all included nine experiments ranged from 1.35 K (0.98 K) to 3.38 K (3.04 K) when considering the individual results from each reader and sensor. The overall median over all experiments, readers, and sensors was 1.85 K (1.82 K).

**Table 1:** RMS-averaged temperature differences Δ*T*_RMS_

Δ*T*_RMS_ (K)	Reader-selected single-voxel ROI						minRMSE single-voxel ROI					
	Reader 1		Reader 2		Reader 3		Reader 1		Reader 2		Reader 3	
	Sens.1	Sens.2	Sens.1	Sens.2	Sens.1	Sens.2	Sens.1	Sens.2	Sens.1	Sens.2	Sens.1	Sens.2
Exp. 1	3.43	1.58	8.55	1.58	3.43	1.58	1.51	0.64	2.18	0.64	1.51	0.64
Exp. 2	9.18	3.58	9.18	1.48	3.61	1.48	2.51	0.81	2.51	0.81	2.51	0.81
Exp. 3	3.38	1.85	3.38	1.85	3.38	1.05	1.25	0.93	1.25	0.93	1.25	0.93
Exp. 5	1.06	0.63	1.06	0.63	1.06	0.63	0.42	0.39	0.42	0.39	0.42	0.39
Exp. 6	2.19	0.46	2.19	0.46	2.19	0.46	0.32	0.21	0.32	0.21	0.32	0.21
Exp. 7	1.49	2.38	1.49	1.35	1.49	1.35	0.60	0.31	0.60	0.31	0.60	0.31
Exp. 8	4.69	0.65	2.97	0.65	2.97	0.65	1.33	0.64	1.33	0.64	1.33	0.64
Exp. 9	5.06	2.99	3.00	1.98	3.00	1.98	0.46	0.41	0.46	0.59	0.46	0.59
Exp. 10	2.68	1.66	5.02	1.66	2.68	1.66	0.90	0.54	0.90	0.54	0.90	0.54
Median	3.38	1.66	3.00	1.48	2.97	1.35	0.90	0.54	0.90	0.59	0.90	0.59
IQR	3.04	2.05	4.95	1.12	1.57	0.98	0.98	0.38	1.32	0.38	0.98	0.38

Data are given in kelvin (K)

*Exp.* Experiment, *IQR* Interquartile range, *minRMSE* Minimum root-mean-squared error, *RMS* Root-mean-squared, *ROI* Region of interest, *Sens.* Sensor

**Fig. 7:**
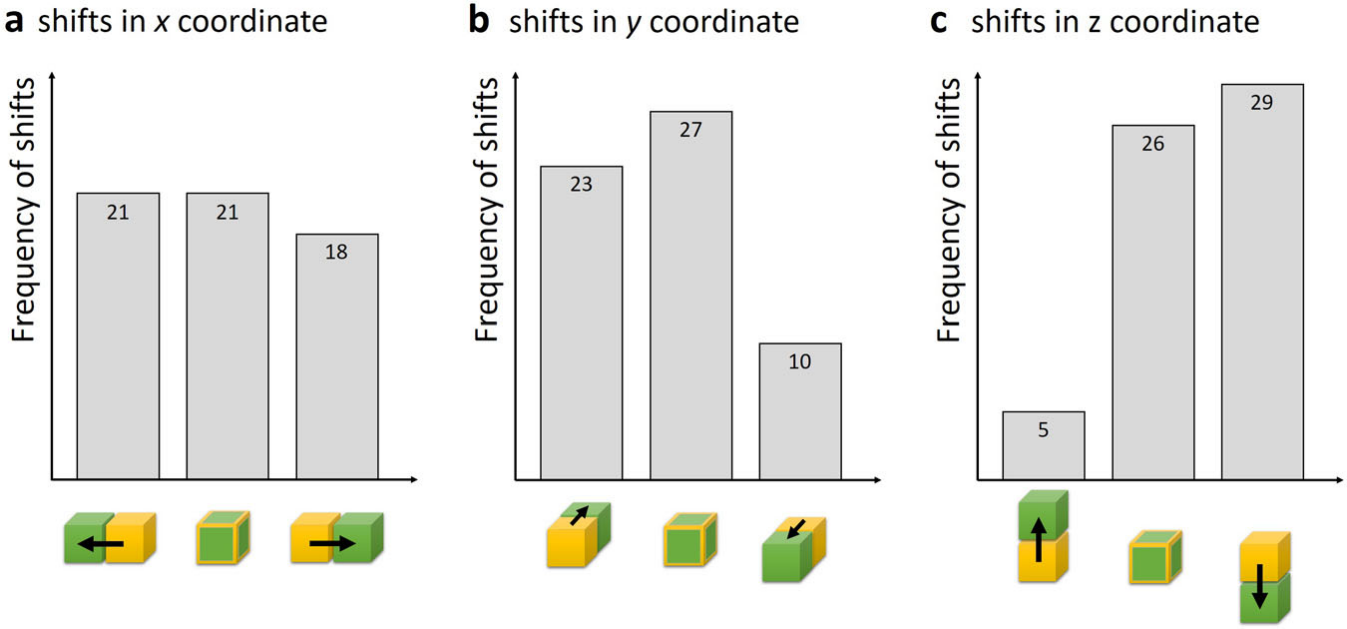
Required shifts of reader-selected voxels to minimize the temperature differences: (**a**) shifts in the *x* direction (within the coronal plane), (**b**) shifts in the *y* direction (within the coronal plane), and (**c**) shifts in the *z* direction (through-plane)

We then evaluated the temperature differences also for 18 voxels neighboring the reader-selected ones and determined the voxel with minimal RMS-averaged temperature difference. As shown in Fig. 5b, there was always a neighboring voxel with a smaller temperature difference; in 14 cases, this was a direct neighbor and in 46 cases a (planar) diagonal neighbor. As shown in Fig. 7, the positions of these minRMSE voxels (relative to the original ones) were relatively isotropically distributed in both in-plane directions, but systematically more often in a deeper (coronal) slice than selected by the readers.

Based on these minRMSE ROIs, the minimal RMS-averaged temperature differences ranged from 0.54 K (0.38 K) to 0.90 K (0.98 K) (see Table 1, right half and Fig. 6b). Systematically higher differences were observed for the sensors that were closer to the microwave antenna. The overall median over all experiments, readers, and sensors was 0.62 K (0.51 K). However, in individual experiments, higher RMS-averaged differences up to 2.5 K were observed. Examples of the MRI thermometry time courses for the minRMSE voxel ROIs of Reader 1 are also shown in Fig. 3 as dashed lines with very good agreement with the reference sensor measurements.

The maximum (positive and negative) deviations between MRI and the values of the reference sensors are listed in Table 2. The median values (over all experiments) were between 0.97 K and 1.24 K for positive deviations and between 0.93 K and 2.02 K for negative deviations; however, in individual experiments, higher (single-timepoint) deviations up to 10 K were observed.

**Table 2:** Maximum positive and negative temperature deviations Δ*T*_max_

	Maximum positive deviation Δ*T*_max,+_						Maximum negative deviation Δ*T*_max,__−_					
	Reader 1		Reader 2		Reader 3		Reader 1		Reader 2		Reader 3	
	Sens.1	Sens.2	Sens.1	Sens.2	Sens.1	Sens.2	Sens.1	Sens.2	Sens.1	Sens.2	Sens.1	Sens.2
Exp. 1	4.07	1.94	0.74	1.94	4.07	1.94	9.82	0.88	10.24	0.88	9.82	0.88
Exp. 2	7.02	2.52	7.02	2.52	7.02	2.52	8.96	1.70	8.96	1.70	8.96	1.70
Exp. 3	1.86	2.86	1.86	2.86	1.86	2.86	3.16	0.01	3.16	0.01	3.16	0.01
Exp. 5	0.97	1.24	0.97	1.24	0.97	1.24	1.22	1.04	1.22	1.04	1.22	1.04
Exp. 6	0.88	0.87	0.88	0.87	0.88	0.87	0.78	0.52	0.78	0.52	0.78	0.52
Exp. 7	0.32	0.51	0.32	0.51	0.32	0.51	1.57	0.94	1.57	0.94	1.57	0.94
Exp. 8	0.60	1.49	0.60	1.49	0.60	1.49	2.69	0.38	2.69	0.38	2.69	0.38
Exp. 9	0.28	0.93	0.97	0.66	0.97	0.66	1.10	0.93	0.95	1.29	0.95	1.29
Exp. 10	1.61	1.12	1.61	1.12	1.61	1.12	2.02	1.43	2.02	1.43	2.02	1.43
Median	0.97	1.24	0.97	1.24	0.97	1.24	2.02	0.93	2.02	0.94	2.02	0.94
IQR	2.51	1.33	1.07	1.46	2.23	1.46	4.90	0.78	4.98	0.91	4.98	0.91

Data are given in kelvin (K)

*Exp.* Experiment, *IQR* Interquartile range

**Fig. 8:**
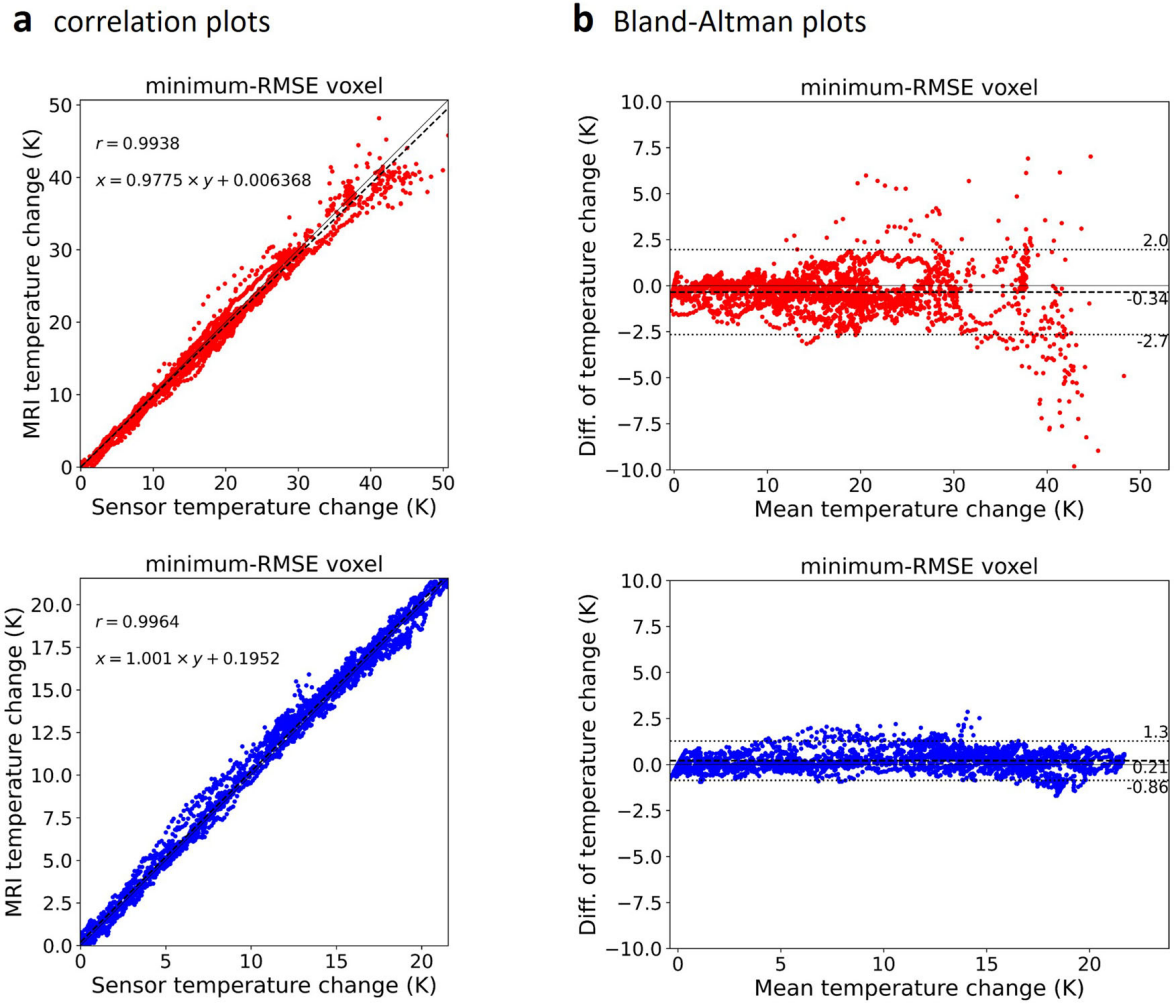
**a** Correlation and (**b**) Bland–Altman plots of MRI-based temperature data (from Reader 1, minRMSE voxels) compared to reference data from fiber-optical sensors (red: sensor closer to microwave antenna, blue: sensor more distant from microwave antenna, note the different scaling of the corresponding axes). The correlation plots contain linear regression parameters (dashed line: linear fit; thin solid line: identity) and Pearson correlation coefficients, *r*. The Bland–Altman plots contain mean differences (dashed line) and 95% confidence limits of agreement (dotted). minRMSE, Minimum root-mean-squared error

Correlation and Bland–Altman plots of data of Reader 1 (representative for all three readers, more results are provided as Supplemental Material) are shown in Fig. 8. Correlation between reference and MRI-based temperatures are highly significant (all *p*-values < 0.0001). Pearson’s correlation coefficient, *r*, was greater than 0.993 for the minRMSE voxel for all readers. The slope of the linear regression line was between 0.956 and 1.001 for the minRMSE voxel. The Bland–Altman plots of Reader 1 show a bias of -0.34 K and +0.21 K for the two sensors, respectively. The limits of agreement are [-2.7 K; 2.0 K] and [-0.86 K; 1.3 K], respectively.

## Discussion

Our results demonstrate that accurate 3D real-time MRI temperature mapping during microwave heating with very low RMS-averaged temperature errors below 1 K is feasible in gel phantoms. The presence of the microwave antenna and the active MRI-conditional in-room microwave generator did not induce any relevant RF artifacts, as demonstrated in Fig. 3 and confirmed by the high accuracy of our MRI temperature measurements. The obtained temporal resolution was 2 s (0.5 Hz) for the acquisition of 3D MRI volumes with 25 slices; the spatial resolution was approximately isotropic with 2.2 × 2.2 × 3.0 mm³.

Potentially, reliable MRI thermometry is highly relevant for thermal local tumor ablation, which—besides resection—represents an important therapeutic option in the treatment of liver tumors [11–16]. Currently, however, therapy planning (*i.e*., setting treatment duration and target temperature or microwave power) is based on static empiric models for the prediction of the expected coagulation zone. The consequences of these predefined treatment settings are higher local recurrence rates and constraints on the application of the technique in proximity to heat-vulnerable structures. Therefore, real-time monitoring of temperature distribution and thermal dose can be expected to improve local tumor control, as well as procedural safety, and might allow the extension of the limits of thermal ablation of tumors in the liver and in other organs. However, the application of *in vivo* real-time MR thermometry is still a work in progress and further improvements regarding reliability, motion correction, temporal solution, and robustness are required.

As illustrated in Fig. 2, the spatial resolution of the EPI acquisition was relatively low in comparison to the 1.15-mm diameter of the fiber-optical sensors, although the sensors appeared considerably larger in the image data due to the surrounding low-signal susceptibility artifact [33, 34]. Consequently, the exact determination of the position of the sensitive sensor tip was challenging, but of high importance for the precise determination of the accuracy of the MRI thermometry data. To overcome this difficulty, three independent experienced readers (two radiologists, and one physicist) defined single-voxel ROIs at the (visually perceived) tip of the sensor in EPI magnitude data. About two-thirds of the defined ROIs (42 of 60 pairwise comparisons, Fig. 5a) agreed exactly between readers, while the remaining third deviated by one voxel (direct or diagonal neighbor), indicating a reasonable accuracy and reproducibility of the subjective ROI definition.

However, the visually perceived sensor tip need not necessarily agree exactly with the actual voxel of our thermometry data that best corresponds to the sensor. This may be due to the appearance of the relatively large susceptibility artifact around the fiber, which was up to three voxels (about 6 mm) wide. In addition, the actual temperature-sensitive volume of the fiber is found “below” the end of the fiber. Therefore, to allow for a remaining disagreement between the MRI-visual fiber tip and the sensor position, we evaluated not only the selected voxels, but also all directly neighboring voxels (18 voxels in a slightly prolate spheroid around the original one) to find the voxel with the best agreement to the reference measurement. The detailed analysis of these additional evaluations showed that it was always possible to find a neighboring voxel, in which MRI thermometry agreed better with the sensor data than in the original voxel (in a RMS-averaged sense). The required shifts were approximately isotropically distributed in the coronal plane (perpendicular to the fiber direction), but substantially more often oriented towards a deeper than a higher slice (29 *versus* 5 shifts). This last observation is in agreement with the position of the sensitive sensor volume below the actual sensor tip. After this additional ROI selection step, the finally selected minRMSE voxel agreed in almost all (56 out of 60, Fig. 5c) pairwise comparisons between the three readers indicating that this procedure mitigated the subjective influences of ROI selection. It should be noted that voxel selection was performed directly on the EPI magnitude images (*i.e*., on data exactly corresponding to the temperature maps, and not on a high-resolution MR acquisition) such that unavoidable EPI-related geometric distortions did not require any correction (but the readers had to work with a relatively low spatial resolution; see Fig. 2).

The resulting median differences, Δ*T*_RMS_, of MRI thermometry and reference measurement were 1.85 K (IQR: 1.82 K) based on the originally defined voxels and 0.62 K (0.51 K) for the minRMSE voxels. Both results present low differences (*i.e*., good accuracy) when compared to the total (median) heating of about 20 K at the sensor positions in the experiments. Similar accuracies between 0.5 °C and 2.8 °C have been reported by Faridi et al [35]. In contrast to the present study, they used a custom-built microwave applicator (instead of a commercially available CE-marked clinical system), and MR thermometry was performed in only three orthogonal slices (instead of a 3D volume as in the present work) [35].

It should be noted that the temperature differences of the MRI thermometry were determined as presented in K (or, equivalently, degrees Celsius, °C) without any additional normalization or calibration factors applied. Thus, the agreement of the measurements as shown in Fig. 3 is not only qualitative (*i.e.*, the curves are not only very well correlated), but quantitative (the values of the two curves agree, as also indicated by the metric Δ*T*_RMS_). This is additionally illustrated by the linear regression analysis with slopes very close to 1 (Fig. 8).

The maximum temperature changes measured by the fiber-optical sensors varied substantially between experiments from 7.3 K to 50.7 K. This was, on the one hand, related to the two different phantoms due to the much more effective heating of the saline-based phantom. On the other hand, these results reflect also slight variations in the distance between the microwave antenna and the sensors (with larger distances corresponding to lower temperature changes). From an experimental point of view, these variations are actually desirable since they allowed us to assess the accuracy in more different setups and parameter ranges than if all sensors had shown exactly the same temperature time course.

This study has limitations. First, all measurements of this study were performed *in vitro* in two kinds of gel phantoms. While this provides improved reproducibility due to the high homogeneity of the phantoms, there are also several effects that are only present *in vivo* and whose influence on the accuracy of MRI thermometry could therefore not be assessed here. In particular, MRI-based temperature measurements *in vivo* are influenced by tissue heterogeneity, motion, macroscopic blood flow, perfusion, and other sources of physiological noise, which must be expected to reduce the accuracy of the measurements. Hence, measurements in phantoms can only provide a best-case estimation of temperature accuracies, whereas accuracies *in vivo* must be expected to be worse.

Second, the two gel phantoms (water and saline-based) exhibited substantially different heating behavior with the water-based phantom showing much less effective heating by the available microwave system than the saline-based one. As a consequence of the much more effective heating, the saline-based phantom showed some effects due to overheating (*i.e*., gel vaporization close to the antenna) manifesting as noisy sensor data (at low overheating as in experiments #1 and #2) or as atypical and invalid MRI thermometry data (as in the discarded experiment #4).

Third, this study was based on only nine heating experiments (after the exclusion of one experiment with the overheated gel) performed at a field strength of 1.5 T. However, the total number of evaluated data points is much higher since each experiment was performed over 700 s with a temporal resolution of 2 s, providing 350 data points for each sensor, *i.e.*, 6,300 data points with evaluable temperature data overall measurements. Different results should be expected at 3 T because of potentially different RF noise interactions between the microwave generator and the MRI system, a different MRI protocol (with, *e.g.*, modified optimal echo time and receiver bandwidth), and potentially larger susceptibility artifacts around the microwave antenna.

Fourth, the spatial resolution of the EPI acquisition was relatively low (2.2 × 2.2 × 3.0 mm³). These voxels are considerably larger than the sensitive volume of the fiber-optical sensors (with a sensitive area diameter of 300 µm), and hence, no perfect agreement between sensor data and temperatures in single voxels can be expected. However, the spatial resolution of the EPI protocol was limited by the required field of view, a feasible matrix size of 128 × 128, and signal-to-noise ratio considerations.

Finally, there may be some influences of the fiber-optical sensors themselves on the accuracy of the MRI thermometry data, potentially worsening the determined accuracy values. The measurement accuracy of the reference sensors can also slightly worsen the measured results. However, since reference data is required for this study, these effects cannot be avoided and it should be noted that the actual accuracies may be even better than the ones determined in our experiments.

In conclusion, accurate 3D real-time MRI temperature mapping during microwave heating with very low typical RMS-averaged temperature errors below 1 K is feasible in gel phantoms, but further evaluation of the accuracy and robustness of MRI-based temperature mapping *in vivo* (*e.g.*, in large-animal studies) is needed.

### Supplementary information

**Additional file 1: Supplementary Fig. S1.** Correlation (a) and Bland-Altman (b) analysis of MRI-based temperature data (**from Reader 1, reader-selected voxels**) compared to reference data from fiber-optical sensors (red: sensor closer to microwave antenna, blue: sensor more distant from microwave antenna, note the different scaling of the corresponding axes). The correlation plots contain linear regression parameters (dashed line: linear fit; thin solid line: identity) and Pearson correlation coefficients, r. The Bland-Altman plots contain mean differences (dashed line) and (95%-confidence) limits of agreement (dotted). **Supplementary Fig. S2.** Correlation (a) and Bland-Altman (b) analysis of MRI-based temperature data (**from Reader 1, minimum-RMSE voxels**) compared to reference data from fiber-optical sensors (red: sensor closer to microwave antenna, blue: sensor more distant from microwave antenna, note the different scaling of the corresponding axes). The correlation plots contain linear regression parameters (dashed line: linear fit; thin solid line: identity) and Pearson correlation coefficients, r. The Bland-Altman plots contain mean differences (dashed line) and (95%-confidence) limits of agreement (dotted). **Supplementary Fig. S3.** Correlation (a) and Bland-Altman (b) analysis of MRI-based temperature data (**from Reader 2, reader-selected voxels**) compared to reference data from fiber-optical sensors (red: sensor closer to microwave antenna, blue: sensor more distant from microwave antenna, note the different scaling of the corresponding axes). The correlation plots contain linear regression parameters (dashed line: linear fit; thin solid line: identity) and Pearson correlation coefficients, r. The Bland-Altman plots contain mean differences (dashed line) and (95%-confidence) limits of agreement (dotted). **Supplementary Fig. S4.** Correlation (a) and Bland-Altman (b) analysis of MRI-based temperature data (**from Reader 2, minimum-RMSE voxels**) compared to reference data from fiber-optical sensors (red: sensor closer to microwave antenna, blue: sensor more distant from microwave antenna, note the different scaling of the corresponding axes). The correlation plots contain linear regression parameters (dashed line: linear fit; thin solid line: identity) and Pearson correlation coefficients, r. The Bland-Altman plots contain mean differences (dashed line) and (95%-confidence) limits of agreement (dotted). **Supplementary Fig. S5.** Correlation (a) and Bland-Altman (b) analysis of MRI-based temperature data (**from Reader 3, reader-selected voxels**) compared to reference data from fiber-optical sensors (red: sensor closer to microwave antenna, blue: sensor more distant from microwave antenna, note the different scaling of the corresponding axes). The correlation plots contain linear regression parameters (dashed line: linear fit; thin solid line: identity) and Pearson correlation coefficients, r. The Bland-Altman plots contain mean differences (dashed line) and (95%-confidence) limits of agreement (dotted). **Supplementary Fig. S6.** Correlation (a) and Bland-Altman (b) analysis of MRI-based temperature data (**from Reader 3, minimum-RMSE voxels**) compared to reference data from fiber-optical sensors (red: sensor closer to microwave antenna, blue: sensor more distant from microwave antenna, note the different scaling of the corresponding axes). The correlation plots contain linear regression parameters (dashed line: linear fit; thin solid line: identity) and Pearson correlation coefficients, r. The Bland-Altman plots contain mean differences (dashed line) and (95%-confidence) limits of agreement (dotted). **Supplementary Table S1.** Correlation, linear regression, and Bland-Altman results in reader-selected ROIs. **Supplementary Table S2.** Correlation, linear regression, and Bland-Altman results in minimum-RMSE ROIs.

## Data Availability

The datasets used and/or analyzed during the current study are available from the corresponding author upon reasonable request.

## Abbreviations

3D: Three-dimensional
EPI: Echo-planar imaging
IQR: Interquartile range
minRMSE: Minimum root-mean-squared error
MRI: Magnetic resonance imaging
RF: Radiofrequency
RMS: Root-mean-squared
ROI: Region of interest
