# Supplementary material for: Accuracy of 3D real-time MRI temperature mapping in gel phantoms during microwave heating
Source: Eur Radiol Exp. 2024 Aug 14;8:92. doi: 10.1186/s41747-024-00479-5 (PMC11324620; doi:10.1186/s41747-024-00479-5)
Supplement: Supplementary file 1 — Additional file 1: Supplementary Fig. S1. Correlation (a) and Bland-Altman (b) analysis of MRI-based temperature data (from Reader 1, reader-selected voxels) compared to reference data from fiber-optical sensors (red: sensor closer to microwave antenna, blue: sensor more distant from microwave antenna, note the different scaling of the corresponding axes). The correlation plots contain linear regression parameters (dashed line: linear fit; thin solid line: identity) and Pearson correlation coefficients, r. The Bland-Altman plots contain mean differences (dashed line) and (95%-confidence) limits of agreement (dotted). Supplementary Fig. S2. Correlation (a) and Bland-Altman (b) analysis of MRI-based temperature data (from Reader 1, minimum-RMSE voxels) compared to reference data from fiber-optical sensors (red: sensor closer to microwave antenna, blue: sensor more distant from microwave antenna, note the different scaling of the corresponding axes). The correlation plots contain linear regression parameters (dashed line: linear fit; thin solid line: identity) and Pearson correlation coefficients, r. The Bland-Altman plots contain mean differences (dashed line) and (95%-confidence) limits of agreement (dotted). Supplementary Fig. S3. Correlation (a) and Bland-Altman (b) analysis of MRI-based temperature data (from Reader 2, reader-selected voxels) compared to reference data from fiber-optical sensors (red: sensor closer to microwave antenna, blue: sensor more distant from microwave antenna, note the different scaling of the corresponding axes). The correlation plots contain linear regression parameters (dashed line: linear fit; thin solid line: identity) and Pearson correlation coefficients, r. The Bland-Altman plots contain mean differences (dashed line) and (95%-confidence) limits of agreement (dotted). Supplementary Fig. S4. Correlation (a) and Bland-Altman (b) analysis of MRI-based temperature data (from Reader 2, minimum-RMSE voxels) compared to reference data fro [file 41747_2024_479_MOESM1_ESM.pdf]

Accuracy of 3D real-time MRI temperature mapping in gel phantoms during microwave heating

ELECTRONIC SUPPLEMENTARY MATERIAL

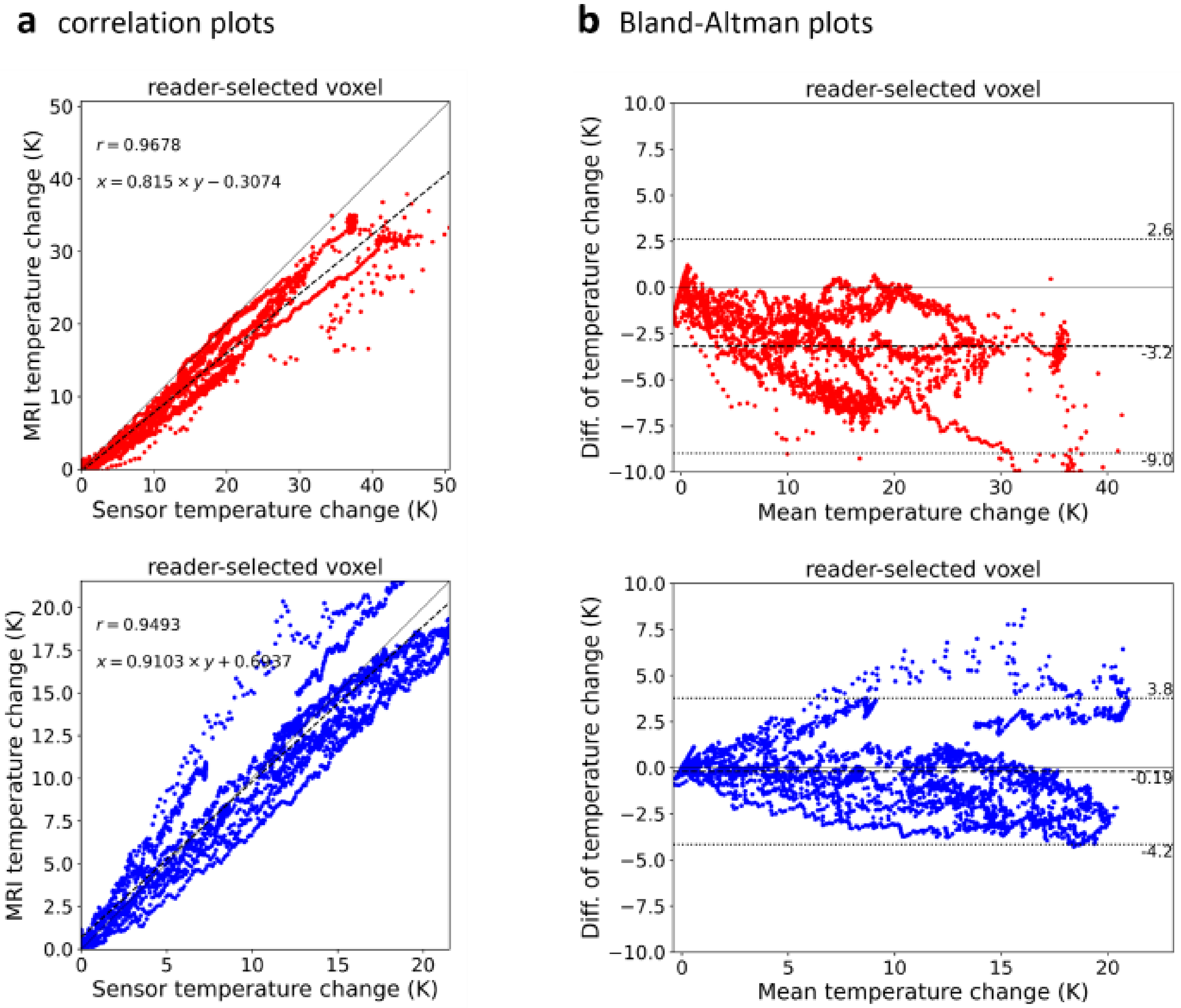

Supplemental Fig. S1. Correlation (a) and Bland-Altman (b) analysis of MRI-based temperature data (**from Reader 1, reader-selected voxels**) compared to reference data from fiber-optical sensors (red: sensor closer to microwave antenna, blue: sensor more distant from microwave antenna, note the different scaling of the corresponding axes). The correlation plots contain linear regression parameters (dashed line: linear fit; thin solid line: identity) and Pearson correlation coefficients, r. The Bland-Altman plots contain mean differences (dashed line) and (95%-confidence) limits of agreement (dotted).

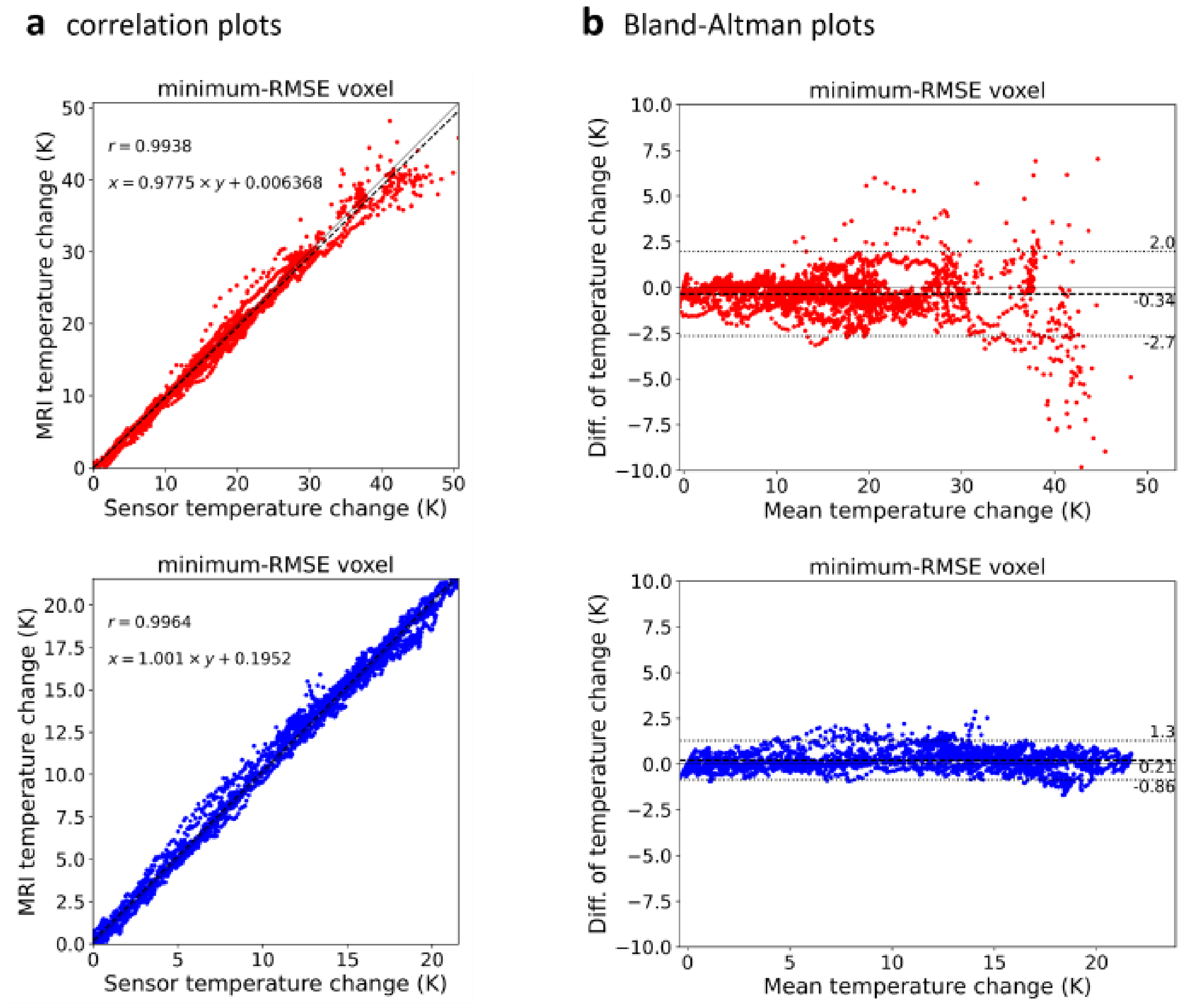

Supplemental Fig. S2. Correlation (a) and Bland-Altman (b) analysis of MRI-based temperature data (**from Reader 1, minimum-RMSE voxels**) compared to reference data from fiber-optical sensors (red: sensor closer to microwave antenna, blue: sensor more distant from microwave antenna, note the different scaling of the corresponding axes). The correlation plots contain linear regression parameters (dashed line: linear fit; thin solid line: identity) and Pearson correlation coefficients, r. The Bland-Altman plots contain mean differences (dashed line) and (95%-confidence) limits of agreement (dotted).

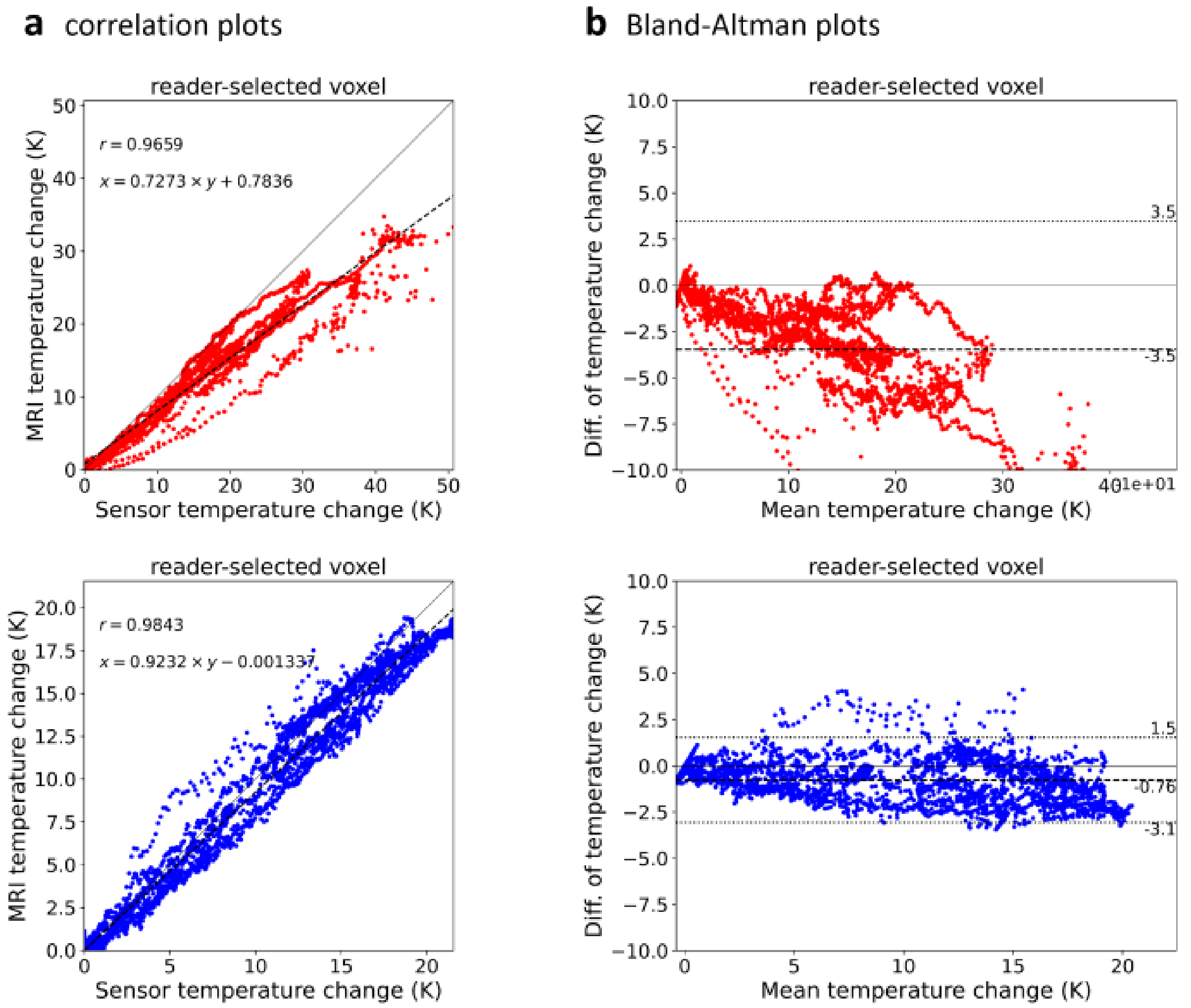

Supplemental Fig. S3. Correlation (a) and Bland-Altman (b) analysis of MRI-based temperature data (**from Reader 2, reader-selected voxels**) compared to reference data from fiber-optical sensors (red: sensor closer to microwave antenna, blue: sensor more distant from microwave antenna, note the different scaling of the corresponding axes). The correlation plots contain linear regression parameters (dashed line: linear fit; thin solid line: identity) and Pearson correlation coefficients, r. The Bland-Altman plots contain mean differences (dashed line) and (95%-confidence) limits of agreement (dotted).

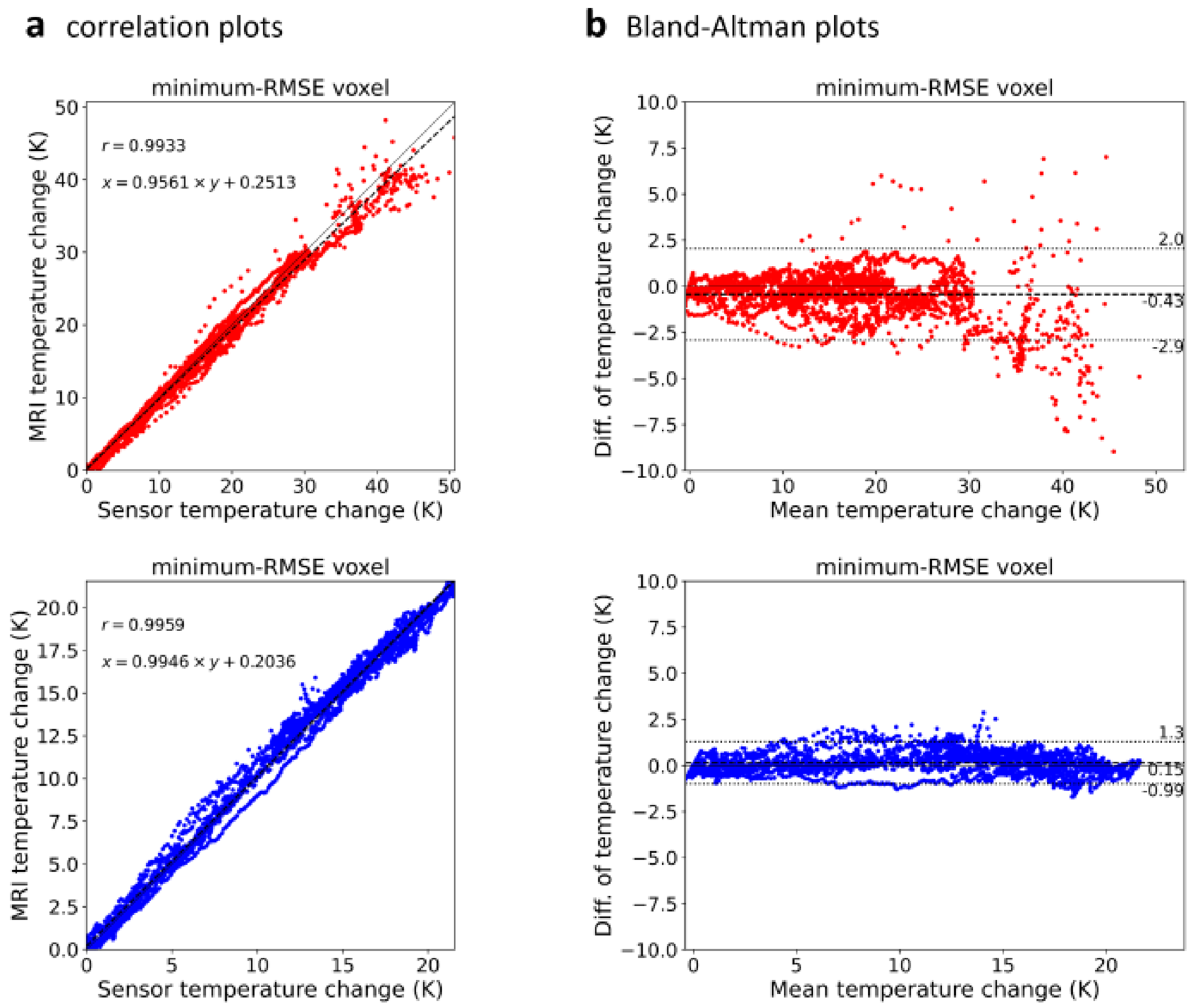

Supplemental Fig. S4. Correlation (a) and Bland-Altman (b) analysis of MRI-based temperature data (**from Reader 2, minimum-RMSE voxels**) compared to reference data from fiber-optical sensors (red: sensor closer to microwave antenna, blue: sensor more distant from microwave antenna, note the different scaling of the corresponding axes). The correlation plots contain linear regression parameters (dashed line: linear fit; thin solid line: identity) and Pearson correlation coefficients, r. The Bland-Altman plots contain mean differences (dashed line) and (95%-confidence) limits of agreement (dotted).

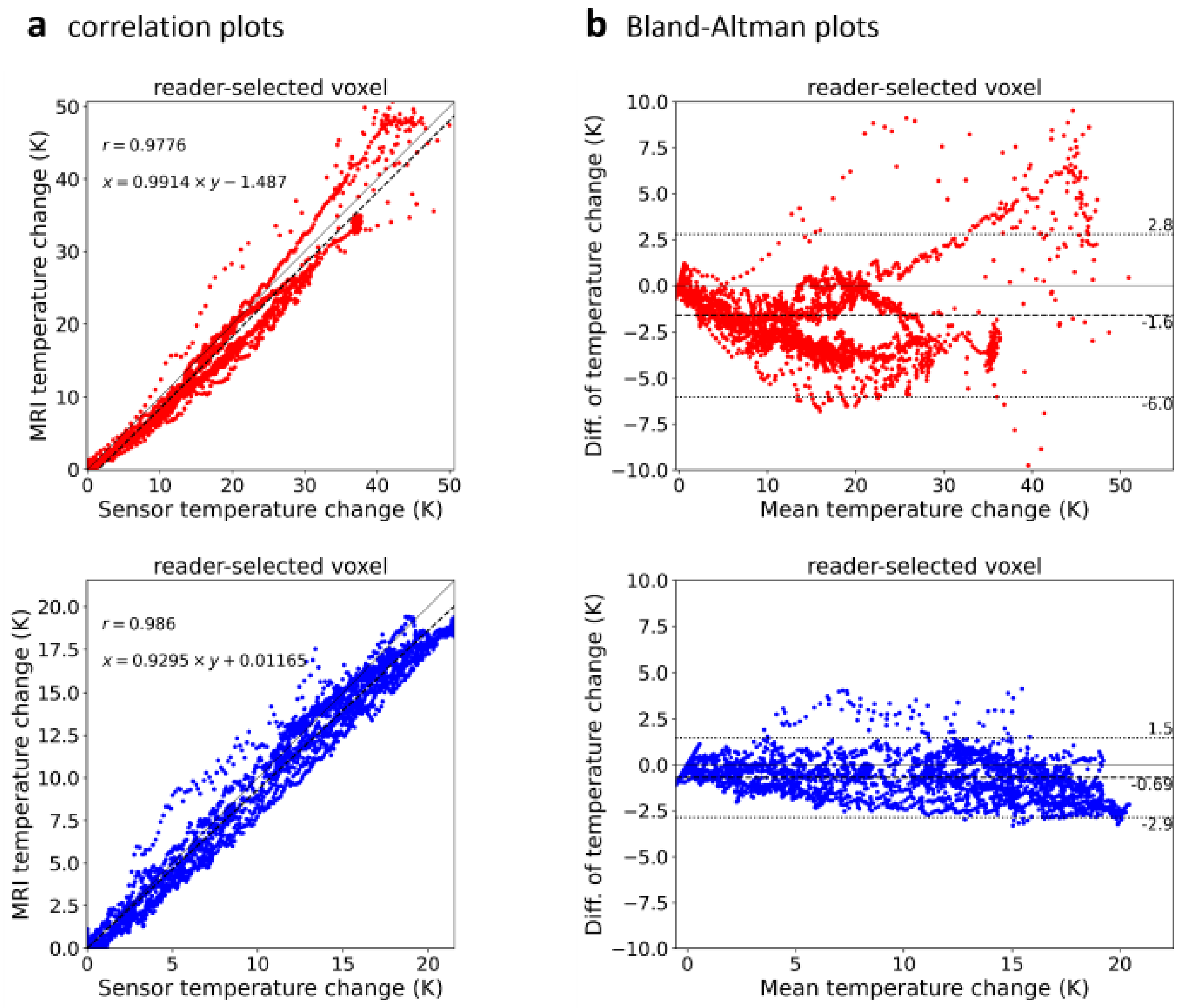

Supplemental Fig. S5. Correlation (a) and Bland-Altman (b) analysis of MRI-based temperature data (**from Reader 3, reader-selected voxels**) compared to reference data from fiber-optical sensors (red: sensor closer to microwave antenna, blue: sensor more distant from microwave antenna, note the different scaling of the corresponding axes). The correlation plots contain linear regression parameters (dashed line: linear fit; thin solid line: identity) and Pearson correlation coefficients, r. The Bland-Altman plots contain mean differences (dashed line) and (95%-confidence) limits of agreement (dotted).

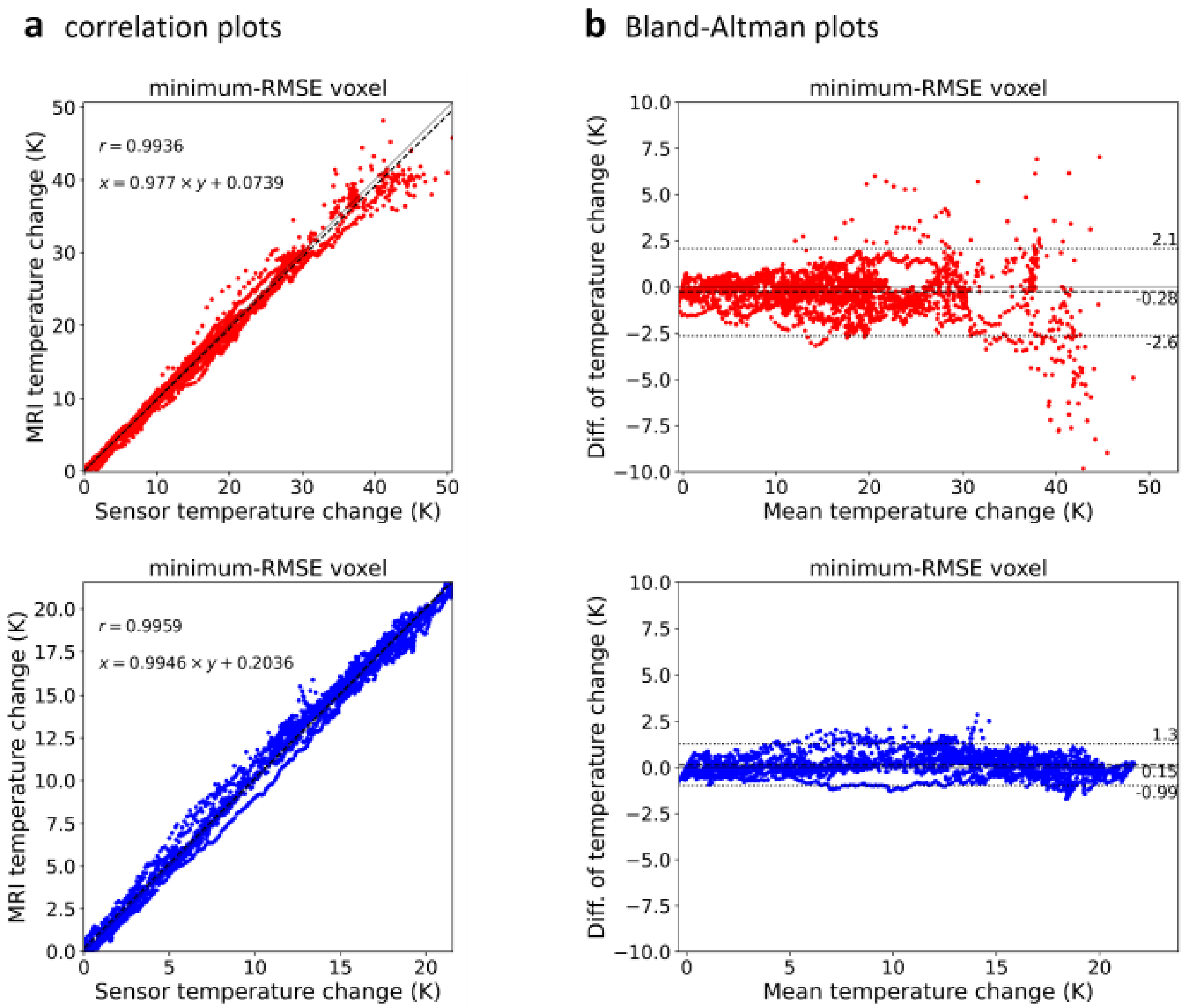

Supplemental Fig. S6. Correlation (a) and Bland-Altman (b) analysis of MRI-based temperature data (**from Reader 3, minimum-RMSE voxels**) compared to reference data from fiber-optical sensors (red: sensor closer to microwave antenna, blue: sensor more distant from microwave antenna, note the different scaling of the corresponding axes). The correlation plots contain linear regression parameters (dashed line: linear fit; thin solid line: identity) and Pearson correlation coefficients, r. The Bland-Altman plots contain mean differences (dashed line) and (95%-confidence) limits of agreement (dotted).

Supplemental Table S1. Correlation, linear regression, and Bland-Altman results in reader-selected ROIs

	Reader-selected (single-voxel) ROI					
	Reader 1		Reader 2		Reader 3	
	Sens.1	Sens.2	Sens.1	Sens.2	Sens.1	Sens.2
Pearson *r*	0.968	0.949	0.966	0.984	0.978	0.986
Linear regression: slope	0.815	0.910	0.727	0.923	0.991	0.929
Linear regression: offset (K)	−0.307	0.694	0.784	−0.001	−1.487	0.012
Bland-Altman mean diff. (K)	−3.183	−0.195	−3.455	−0.762	−1.621	−0.687
Bland-Altman lower limit (K)	−8.996	−4.158	−10.382	−3.062	−6.037	−2.860
Bland-Altman upper limit (K)	2.631	3.768	3.472	1.538	2.796	1.487

Supplemental Table S2. Correlation, linear regression, and Bland-Altman results in minimum-RMSE ROIs

	Reader-selected (single-voxel) ROI					
	Reader 1		Reader 2		Reader 3	
	Sens.1	Sens.2	Sens.1	Sens.2	Sens.1	Sens.2
Pearson *r*	0.994	0.996	0.993	0.996	0.994	0.996
Linear regression: slope	0.978	1.001	0.956	0.995	0.977	0.995
Linear regression: offset (K)	0.006	0.195	0.251	0.204	0.074	0.204
Bland-Altman mean diff. (K)	−0.343	0.209	−0.431	0.150	−0.284	0.150
Bland-Altman lower limit (K)	−2.653	−0.860	−2.909	−0.991	−2.625	−0.991
Bland-Altman upper limit (K)	1.967	1.279	2.047	1.291	2.057	1.291
